# You are what you eat: autophagy guides CD8^+^ T cell function through metabolism

**DOI:** 10.1097/IN9.0000000000000064

**Published:** 2025-06-26

**Authors:** Caio Loureiro Salgado, Henrique Borges da Silva

**Affiliations:** 1 Department of Immunology, Mayo Clinic, Phoenix, AZ, USA; 2 Department of Cancer Biology, Mayo Clinic, Phoenix, AZ, USA

**Keywords:** autophagy, CD8^+^ T cells, cytotoxic T cell, naive T cells, mitophagy

## Abstract

The differentiation of naive CD8^+^ T cells into effector or memory populations requires dynamic remodeling of cellular metabolism and proteome composition. In a recent study published in *Nature Immunology*, Sinclair et al offer critical insights into the role of autophagy, particularly mitophagy, in regulating these processes during CD8^+^ T cell differentiation. Autophagy, a conserved catabolic mechanism, is traditionally associated with cellular homeostasis and survival during nutrient deprivation. In contrast, Sinclair et al reveal that, in the immune system, autophagy is not simply a survival mechanism but a fine-tuned regulator of CD8^+^ T cell metabolism and function, fine-tuning CD8^+^ T cell effector vs quiescence choices.

CD8^+^ T cells must dynamically regulate their intracellular pathways to transition between their different activation states. Naive and memory CD8^+^ T cells are relatively quiescent, displaying low levels of protein synthesis and low proliferative capacity. In contrast, effector CD8^+^ T cells have high proliferative capacity and increased protein synthesis ^[1]^. Protein synthesis not only depends on mRNA transcription but also on extrinsic amino acid sources. Those can be either obtained from the extracellular microenvironment through transmembrane transporters ^[2]^ or alternatively recycled through autophagy, an intracellular pathway that degrades proteins into amino acids. Autophagy has been indicated to play important roles in the promotion of naive and memory CD8^+^ T cell survival ^[3,4]^ while dampening the effector activity of CD8^+^ T cells in tumor contexts ^[5]^. However, these studies contrast with other findings suggesting that autophagy proteins such as MAP1LC3b are upregulated in effector T cells, if compared to naive or memory T cells ^[6]^.

To solve this conundrum, Sinclair et al, in a recent paper published in *Nature Immunology* ^[7]^, used a combination of autophagic flux measurements: a dynamic reporter based on acid pH GFP quenching and mass spectrometry. Autophagic flux is commonly defined as the measuring of autophagic organelle degradation activity rate ^[8]^, and the authors reasoned that tracking the activity of autophagy will paint a more precise picture of the usage of this pathway, rather than static markers. Using these readouts, the authors showed that naive CD8^+^ T cells exhibit high basal autophagic flux. This activity appears to be functionally linked to mitophagy, the selective degradation, or trimming of abnormal mitochondria, which in turn sustains mitochondrial quality control, supports cellular migration, and ensures survival in a resting state. More specifically, the authors found that increased mitophagy in T cells was correlated with the maintenance of a naive phenotype (eg, higher expression of CD62L and lower expression of CD44) in CD8^+^ T cells. Conversely, exposure to T cell receptor (TCR)-specific signals and IL-2 led to a drop in autophagic flux. The inhibition of autophagy was followed by increased proliferation, increased expression of glucose and lactate transporters, and glycolysis-mediated adenosine triphosphate (ATP) production. These effects are accentuated in conditions of amino acid deprivation, which leads to increased autophagy levels even in activated CD8^+^ T cells. In contrast with IL-2, which drives the effector phenotype in CD8^+^ T cells, exposure to the memory-associated cytokines IL-15 or IL-7 maintains autophagic flux levels high. This correlates with the importance of autophagy in the induction of memory CD8^+^ T cells and overall CD8^+^ T cell survival, including in the presence of amino acid starvation as found by the authors. These findings resonate with earlier observations that autophagy is essential for maintaining T cell homeostasis ^[3,4]^ and suggest that the naive state is intrinsically dependent on catabolic activity to maintain proteostatic balance.

Upon TCR engagement and exposure to inflammatory cytokines such as IL-2 and IL-12, autophagic flux is rapidly and profoundly repressed. This is accompanied by an increase in the expression of autophagy-related genes, a paradox resolved by the observation that active autophagy leads to the degradation of these very components. As such, the accumulation of autophagy proteins in effector T cells reflects reduced flux rather than enhanced function. The suppression of autophagy occurs within hours post-activation, suggesting that it is an early and decisive event in T cell differentiation. These findings challenge previous assumptions that T cell activation would induce autophagy and highlight the necessity of using dynamic flux measurements instead of static markers like LC3B or p62 ^[9]^ (Figure 1A).

**Figure 1.**
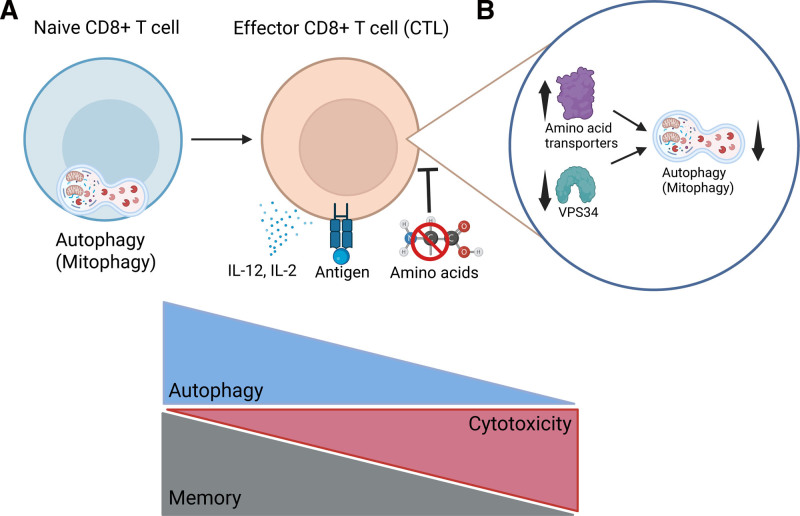
**Autophagy repression is necessary for the effector differentiation of CD8**^+^ **T cells.** (A) Naive CD8^+^ T cells display enhanced levels of autophagic function, more specifically high levels of mitophagy. This function is necessary for the survival of these cells, as well as for the maintenance of their metabolic and functional quiescence (ie, low protein synthesis and cytotoxic function). Upon TCR priming and sensing of pro-inflammatory cytokines such as IL-2 or IL-12, CD8^+^ T cells downregulate their autophagic flux. This downregulation is not only correlated but is necessary for CD8^+^ T cells to increase their proliferation and effector function. Memory CD8^+^ T cells, in contrast, revert to an increase in autophagic flux capacity, at the expense of expression of cytotoxic or other effector molecules. Importantly, these changes are better tracked using autophagic flux function assessments rather than expression of autophagy proteins – which are usually degraded during autophagic function. (B) The repression of autophagic function in effector CD8^+^ T cells not only correlates but relies on the increased availability of amino acids in the extracellular environment and the expression of amino acid transporters such as SLC7A5. This leads to an increase in intracellular amino acid levels, which directly contribute to the repression of autophagic flux. Conversely, the enzyme VPS34, which is crucial for autophagy initiation, is inhibited in effector CD8^+^ T cells with abundant amino acid levels. TCR, T cell receptor. This figure was prepared using BioRender.

Crucially, they identify amino acid availability and transporter expression as major regulators of autophagic flux. During activation, CD8^+^ T cells upregulate amino acid transporters, particularly SLC7A5, SLC1A5, and SLC7A1, enhancing intracellular amino acid abundance and thereby contributing to the suppression of autophagy (Figure 1B). When extracellular amino acids are restricted, activated CD8^+^ T cells revert to high autophagic flux, suggesting a compensatory mechanism to maintain functional capacity. This dynamic adaptation emphasizes the dual role of autophagy in both nutrient recycling and metabolic support during immune activation, aligning with the broader concept of autophagy as a metabolic homeostatic regulator ^[10]^.

Interestingly, the functional consequences of autophagy repression in activated cytotoxic T lymphocytes (CTLs) extend to the proteome itself. Proteomic profiling reveals that in CTLs, autophagy targets not only cytolytic effector molecules such as granzyme B but also nutrient transporters and adhesion proteins. By preventing the degradation of these molecules, autophagy repression reinforces the acquisition of a cytotoxic phenotype. This effect is consistent with findings from tumor models where autophagy was reported to limit antitumor immunity ^[5]^, suggesting a negative role of autophagy in CD8^+^ T cell cytotoxic function.

The authors also examined the role of mitophagy in shaping mitochondrial mass and metabolic configuration. Naive T cells exhibit selective mitochondrial degradation, which is curtailed upon activation, resulting in increased mitochondrial mass and enhanced oxidative phosphorylation. This shift is consistent with previous studies showing that effector T cells rely on mitochondrial activity for their initial activation ^[11]^. Moreover, VPS34, a key autophagy-initiating kinase, is required for maintaining autophagy in naive cells, but its inhibition is tolerated in activated CTLs in nutrient-replete conditions, suggesting distinct requirements for autophagy across different T cell states (Figure 1B). This, however, was not true in nutrient-deprived conditions, where autophagy preserves the expression of reactive oxygen species (ROS)-protecting enzymes. This highlights the nuanced role of autophagy for T cells in conditions of amino acid scarcity such as CTLs infiltrating tumors ^[12]^, since despite a decrease in cytotoxicity, autophagy can also lead to survival.

The use of Bafilomycin A1 (BafA1), a compound that blocks lysosomal v-ATPases, uncovered an additional layer of control over autophagy. In naive CD8^+^ T cells, BafA1 reduced autophagic flux, highlighting the essential role of v-ATPases and induction of lysosomal acidification in sustaining this process. In contrast, BafA1 can also enhance memory T cell responses due to v-ATPase and mTORC1 negative regulation ^[13]^. This may suggest that lysosomal v-ATPases may promote opposite CD8^+^ T cell fates in a context-dependent way, with perhaps the induction of autophagic activity being a rheostat for this. In vivo, autophagic repression was also observed in antigen-experienced, but not in uninfected control CD8^+^ T cells. Furthermore, PD1^+^ tumor-infiltrating CD8^+^ T cells exhibited higher autophagic flux compared with PD1^−^. Given that PD1 expression is a marker of exhaustion, this suggests that sustained autophagy may be associated with dysfunctional or metabolically exhausted T cells in chronic conditions, aligning with reports that autophagy hinders CD8^+^ T cell cytotoxic function against tumors ^[5]^. Whether different modalities of autophagy will have effects on distinct subsets of exhausted CD8^+^ T cells remains to be investigated; a recent study suggests that mitophagy can promote superior antitumor CD8^+^ T cell responses ^[14]^. The effect of autophagy on PD1^+^ CD8^+^ T cells can have strong implications for immunotherapy, where the transient inhibition of autophagy may have synergistic effects with anti-PD1 treatments in exhausted CD8^+^ T cells. Such efforts, however, must be investigated carefully in future studies due to the nuanced effect of autophagy on T cells inside the amino acid-starved tumor microenvironment ^[12]^.

In conclusion, Sinclair et al provide compelling evidence that autophagy is not merely a housekeeping function in CD8^+^ T cells but a dynamically regulated pathway that determines cell fate, effector function, and metabolic programming. Their findings bridge existing gaps between transcriptional, proteomic, and metabolic studies in T cell biology and offer new perspectives on how nutrient sensing and catabolic processes influence adaptive immunity. By uncovering how antigen and cytokine signaling converge to suppress autophagic flux, the study positions autophagy as a critical checkpoint in effector T cell differentiation, with potential implications for the development of immunotherapeutic strategies that modulate autophagy to enhance antitumor or antiviral immunity. For instance, the inhibition of autophagic flux can be re-purposed to increase the cytotoxic potential of CD8^+^ T cells used in adoptive cell therapies. These treatments must be tightly controlled, due to the nutrient scarcity of the tumor microenvironment ^[12]^ that can render CD8^+^ T cells sensitive to autophagy inhibition. Conversely, activation of autophagic flux can be used to boost memory CD8^+^ T cell responses, as suggested by previous works ^[3]^, to induce better vaccination efficacy. This present work, notably, shows the importance of timely control such activation to avoid dampening the initial activation of CD8^+^ T cells.

## Conflict of interest

The authors declare that they have no conflicts of interest.

## Acknowledgments

We thank the members of the Borges da Silva lab for discussion. This manuscript was supported by the grant R01AI170649-03 (H.B.S.).
